# Enhanced Oil Binding Potential of *Procambarus clarkii* Chitosan (PCC): A Study with Extra Virgin Olive Oil and Sunflower Oil Under Simulated Gastric Conditions

**DOI:** 10.3390/polym17111445

**Published:** 2025-05-23

**Authors:** Claudio Casella, Umberto Cornelli, Santiago Ballaz, Giuseppe Zanoni, Luis Ramos-Guerrero

**Affiliations:** 1 Department of Chemistry, University of Pavia, 27100 Pavia, Italy; icarocus@gmail.com (C.C.); gz@unipv.it (G.Z.); 2 School of Medicine, Loyola University, Chicago 60660, IL, USA; ucornelli@gmail.com; 3 Faculty of Health Sciences, Universidad del Espiritu Santo, Samborondón P.O. Box 09-01-952, Ecuador; sballazg@gmail.com; 4 Grupo de Investigación en Bio-Quimioinformática, Carrera de Ingeniería Agroindustrial, Facultad de Ingeniería y Ciencias Aplicadas, Universidad de Las Américas (UDLA), Quito 170513, Ecuador

**Keywords:** chitosan, extra virgin oil, *Procambarus clarkii*, sunflower oil, oil-binding capacity, hydrogel

## Abstract

Chitosan is recognized by its capacity to bind lipids based on the viscosity and degree of deacetylation. We analyzed the in vitro binding of *Procambarus clarkii* chitosan (PCC) with extra virgin olive oil and sunflower oil at temperatures and pH levels that approximate gastric-like conditions. In the tube test, 4 mg of PCC and 0.3 g of either EVO or of SO oils were mixed by stirring in test tubes with 4 mL of water acidified with HCl to a pH of 3. The PCC binding capability was determined by measuring the differences between the suspension without PCC and the suspension with PCC added. A scanning electron microscope (SEM) was utilized to further observe the uniformity and morphology of the emulsified PCC/oil hydrogels. In the test tube, PCC was shown to have a 1:11 (*w/w*) binding capacity for EVO and 1:15 (*w/w*) for SO. The SEM-based examination demonstrated a smooth surface with fine porosity of the microstructure of either PCC/oil hydrogel, proving successful emulsification. Under conditions similar to those in the stomach after a meal, including acidity, mixed composition, and temperature, PCC efficiently binds and emulsifies EVO and SO.

## 1. Introduction

Chitosan is a natural cationic polysaccharide composed of randomly distributed β-(1→4)-linked D-glucosamine and *N*-acetyl-D-glucosamine units. Chitosan is the product of the deacetylation of the chitin present in the exoskeleton of arthropods (crabs, shrimps, lobsters, etc.), and in the cell walls of most fungi and some algae. All forms of chitosan have the ability to bind lipids after deacetylation because of the presence of NH_3_+ charges on their glucosamine monomers, hydrophobic lipid–chitosan interactions, and hydrogen bonding between them [1]. The entity of the binding seems to be inversely proportional to the degree of polymerization (DP) and the degree of deacetylation (DDA) [2,3]. However, this theory is still up for debate because different types of chitosan may have unique properties. For instance, the inverse association with fatty acids appears to be favored when the DDA is more than 90%. Some evidence suggests that chitosan with a very high molecular weight (MW = 810 KDa) and 87% to 96% DDA shows substantially stronger fat-binding (1.5 and 2 times, respectively) than when the DDA is 75% [4]. Furthermore, a medium MW chitosan with a DDA >90% works well with soybean oil at pH 6.8 [5]. However, attempting acid pH ranges is the best way to evaluate chitosan activity, since the gastric environment should be the most important starting point. Some scientists have calculated fat binding in acidic environments, such as the stomach, in vitro using chitosan with a DDA < 90%, [6]. They discovered that when the MW was 30 KDa (5.4 g) instead of 890 KDa (11.07 g), the trapping of soybean oil was much lower (by roughly half) at a 1:40 mass ratio of chitosan/oil.

**Figure 1:**
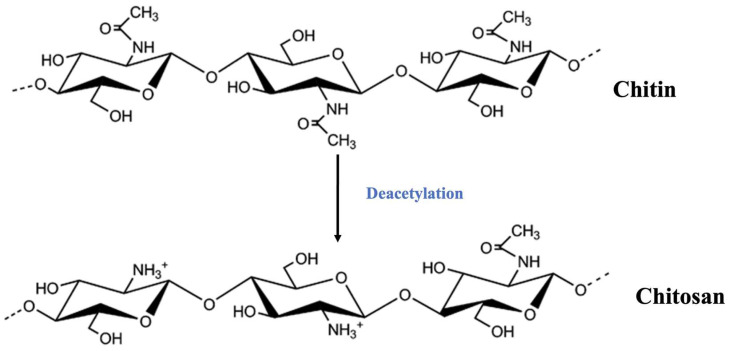
Chitosan dimers are composed of deacetylated and acetylated glucosamine units.

Because every chitosan is commonly used to reduce body weight, fat mass, abdominal girth, and insulin resistance, all chitosan used in humans is evaluated for its ability to bind with lipids. This also applies to chitosan extracted from *Procambarurs clarkii*, also known as PCC (Figure 1), which is classified as a medium-molecular-weight chitosan (MMWC) and has polymers with a viscosity of 100–120 cPs and a MW of 40 to 100 KDa. When taken orally, chitosan must pass through three distinct compartments: the stomach (pH of 2–3), small intestine (duodenum (pH of 6.8), jejunum, and ileum (pH of 7–7.5)), and colon (pH of 7). The stomach is where the pH variation is most obvious.

The majority of the physicochemical characteristics of the chitosan will be ascertained here, where it will be dissolved in the presence of pepsin and gastric acid [7]. Although the cause is unknown, all of the chitosan may be partially hydrolyzed under these circumstances. The pH rises as the chitosan-containing chime bulk enters the small intestine, which is home to pancreatic fluid and bile, forming a sort of gel structure made up of tiny networks of chitosan and other food components like fats, starch, and vitamins. This kind of gel decreases the possibility that lipids will be transported into micelles and absorbed by the enterocyte [8]. The colon is where bacteria use chitosan as probiotic, which reduces its excretion in feces.

Oil/chitosan combinations in neutral pH, centrifugation, and supernatant removal are the commonly used technique to assess the fat-binding capacity (FBC) of chitosan [9]. Since centrifugation and a neutral pH are not what happen in the human stomach, we decided on a more straightforward method that simply uses HCl in solution. Two oils, extra virgin olive oil (EVO) and sunflower oil (SO), were used in relation to their different compositions of monounsaturated (MUFAs), polyunsaturated (PUFAs), and saturated (SAFAs) fatty acids. Whilst PUFAs predominate in the SO, a greater quantity of MUFAs is present in the EVO. Most of the fatty acids (FAs) present in foods are similar to EVO and SO. Over 90% of FAs in these oils are linked to glycerol as trioleins. The majority of trioleins are hydrolyzed in an in vitro acid environment, and the FA/glycerol separates for the two oils in a comparable manner. FA polarity, which rises with the amount of C=C bonds and falls with the number of carbon atoms, determines how much of an affinity they have for chitosan. This theoretically means that PUFAs and oxidized lipids should have a higher affinity for chitosan than SAFAs.

The interaction of chitosan from unconventional sources (PCC with a DDA > 90%) with edible oils (EVO and SO) still is an unexplored area of investigation that may provide a novel contribution to the field. As a goal, this is the first study to investigate whether chitosan (DDA > 90%) could bind EVO and SO in an acidic environment (pH = 3) similar to the stomach using aggregation and SEM image analysis. Because of its well-established bioadhesive and lipophilic interactions, this study’s findings may therefore suggest using chitosan to regulate fat digestion and absorption. This would improve dietary changes and cholesterol-lowering strategies.

The use of PCC as an oil-binding agent in conditions that resemble the gastric environment may open new avenues for the development of oil delivery systems and their possible application in food technology, pharmacy, and medicine. Additionally, by comparing their effectiveness with the characteristics of EVO and SO, this study provides more insights into the functional properties of chitosan and its potential applications in the development of innovative products.

## 2. Materials and Methods

### 2.1. Reagents

*Procambarus clarkii* chitosan or PCC (Nutratrade, Golden Shell, Taizhou, China) is a medium MW chitosan (from 40 to 140 KDa) with a degree of deacetylation of 90% and cPs ranging from 90 to 130. High-quality, commercially available SO (Coosol, Vilches, Spain) was purchased from a Spanish supermarket (KOH value of 0.16), while EVO (Monini, Spoleto, Italy) was acquired from an Italian supermarket (KOH value of 0.16). The oils were sent to Dr. Claudio Casella’s laboratory to determine the distribution of free fatty acids (EVO: 92 g of total fatty acids, including 14.7 g of saturated fatty acids, 73 g of monounsaturated fatty acids, and 7.5 g of polyunsaturated fatty acids; SO: 100 g of total fatty acids, including 13 g of saturated fatty acids, 24 g of monounsaturated fatty acids, and 63 g of polyunsaturated fatty acids). To avoid contamination by pollutants present in the laboratory, all bifiltered distilled water and reagents like HCl at 36% (Sigma-Aldrich, Saint Louis, MO, USA) were first bifiltered through a glass microfiber filter (0.7 μm pore size, Whatman, Florham Park, NJ, USA).

### 2.2. Lipid, Chitosan, and Water Amounts in a Standard Meal

According to the Food Intake Assessment (FIA) system, the quantity of fat consumed during a standard meal of 1200 Kcal is 43 g, of which less than 100 mg is cholesterol [10]. It is estimated that 0.8 g of PCC should be administered prior to meals for 43 g of fat. During a meal, the stomach contains 740 mL of liquid (0.5 L of gastric fluid, 120 mL of water, and 120 mL of wine), However, a significant portion of this is unavailable because of the food and mineral adsorption of water. The fluid available for the chitosan/FA reaction was then experimentally determined to be 0.5 L.

### 2.3. Test Tube Assays

PCC was firstly added to an acidic solution of HCl in water at pH 3 and 36 °C at a concentration of 1.6 mg/mL and then the mixture was continuously stirred for 30 min. Next, 3 g of oil (EVO or SO) was dispensed in a 4 mL cubic tube together with 3 mL of PCC solution (1.6 mg/mL). The PCC/oil blend was stirred for 10 s right after preparation and three more times at 5 min intervals with the aid of vortexing. As a control, the same method was utilized to stir a pH 3 solution of HCl that included either EVO or SO and no PCC. For the pH measurement (in triplicate), a basic benchtop pH meter (ORP sensION^+^ PH3, HACH, Düsseldorf, Germany) was used. The binding capacity of chitosan was indicated by the difference between the PCC and Control suspensions. The following formula was used in quantitative terms: Control/3 = PCC/x. The binding of 1 g of PCC with the grams of either EVO or of SO was then obtained by dividing the x value by three. Eight replicates were conducted for every type of oil.

### 2.4. Aggregate Formation (Laboratory Trials) and SEM Morphological Analysis

A total of 40 mg of PCC and 0.3 g of oil (EVO or SO) were blended in 3 mL of distilled water acidified with HCl at pH 3. A simple benchtop pH meter (ORP sensION^+^ PH3, HACH, Düsseldorf, Germany) was used to monitor the pH (in triplicate). Mixtures were then vortexed for 5 min and subsequently stirred for 30 min at room temperature. After that, samples were left undisturbed for an additional 30 min. Finally, samples were recovered using vacuum filtering with glass microfiber filters (Whatman filter, 0.7 mm pore size, Whatman, Florham Park, NJ, USA). Eight replicates were conducted for each oil.

A semi-automated stereomicroscope (Leica M205FA, Leica Microsystems CMS GmbH, Wetzlar, Germany) equipped with a high-resolution digital color camera (Leica DFC310FX; 1.4 Mpixel, CCD; zoom from 7.85 to ×161, Leica Microsystems CMS GmbH, Wetzlar, Germany) was used to inspect the formation of hydrogel aggregates in the acquired filters. A scanning electron microscope (SEM) (JEOL 6610 LV, JEOL Ltd., Tokyo, Japan) with microanalysis and a secondary electron detector was used to obtain micrographs of the filtered hydrogels. An analysis of the intensity distribution of the aggregates and droplet size in aggregates of the hydrogel was estimated using ImageJ software (Confocal UniOvi ImageJ, LAS V4.0 Leica Application Suite, Version 4.0.0, Leica Microsystems CMS GmbH, Wetzlar, Germany; https://imagej.net/nih-image/ (accessed on 10 January 2025)).

### 2.5. In Vitro Fat-Binding Capacity

Using two fatty materials (EVO and SO), in vitro fat-binding studies were conducted at different pH levels to simulate the human gastric system (without enzymes) and then at a fixed pH of 3.0 (stomach pH). These trials relied on the development of an oil/PCC hydrogel at various pH levels (pH: 3, 4, and 5) [5]. The following approach was taken: To solubilize the PCC, 20 mL of a 0.1 mol/L HCl solution (Sigma-Aldrich, Saint Louis, MO, USA) was mixed with 0.1 g of each PCC sample. The mixture was then stirred for 30 min at 200 rpm and 37 °C. In order to mimic the pH of the human stomach, the pH at this point was 3.0. EVO/SO was gradually added to the PCC solution at a mass ratio of 1:40, or 0.1 g of PCC/4.0 g. The PCC/EVO and PCC/SO emulsions were produced by stirring the mixture in a simulated stomach environment for 30 min at 200 rpm and 37 °C. To replicate the gastric (pH = 3) and duodenal (pH = 4–5) conditions, when partially digested material exits the stomach and enters the duodenum, a controlled volume of 15 g/L of NaHCO_3_ (Sigma-Aldrich, Saint Louis, MO, USA) solution was added to the PCC/EVO and PCC/SO hydrogel to achieve pH values of 3.0, 4.0, and 5.0. A pH of 3.0 was selected in order to replicate the stomach pH, which is primary site of cross-linking between PCC and oils and the goal of the current investigation. The ability of PCC (EVO and SO) to bind fat was compared at this pH value. After 60 min of stomach residence time simulation, the samples were centrifuged for 30 min at 4000 rpm. Using a vacuum filter, the precipitate was filtered. After extracting the oil contained in the PCC gel using 10 mL of ethyl ether (Sigma-Aldrich, Saint Louis, MO, USA), the mixture was vortexed for 30 s using a Vortex (Vortex Shaker, 4000 rpm, Oviedo, Asturias, Spain). The residual oil was extracted from the samples using filtration equipment and 20 mL of ethyl ether (Sigma-Aldrich, Saint Louis, MO, USA). A rotary evaporator (Kintek, model KRE-2011, Zhengzhou, China) was used to recover the entire oil, and any leftover solvent was eliminated by drying it in an oven set at 80 °C for 24 h. The mass of oil trapped in the PCC gel was determined by gravimetric analysis and the fat binding data were expressed as g of oil/g of PCC. Every experiment was conducted in triplicate.

### 2.6. Quality Assurance and Quality Control (QA/QC)

From sampling to quantification, quality assurance and control (QA/QC) processes were carried out using methods described in the literature. Important QA/QC practices included the use of glass microfiber filters (0.7 µm pore size), avoiding polymeric materials in the laboratory, and filtering chemical reagents before use. From sampling to analysis, good field and laboratory procedures (GLP) were followed to minimize the exogenous contamination found in the air, on surfaces, and ultimately on the equipment. Therefore, procedural blanks were used. Control experiments were performed in both situations to avoid any interference with the analyses. Lastly, every experiment and analysis were carried out in triplicate. All chitosan and oil sample filtration procedures were conducted under strict supervision in a certified laminar flow cabinet. In order to prevent sample contamination, this kind of cabinet offers an ISO 5 clean air working environment with a unidirectional flow of sterile, particle-free air. This strategy is generally acknowledged as a component of contamination reduction techniques. The following extra safety measures were noted in addition to operating in laminar flow:

- Prevent the operator or clothing from generating particles by using lint-free lab coats, face masks, and pre-washed nitrile gloves. Before each use, it is advised to pre-wash all lab equipment, such as forceps, funnels, and filters, using Type I ultrapure water. Laminar flow should then be used to dry any leftover residue.
- Blank controls—to evaluate any possible unintentional contamination during the process, procedural blanks, or control filters devoid of samples, were run alongside the experimental samples.
- To reduce their exposure to the environment, always keep the filters in closed or covered systems.

### 2.7. Statistics

When convenient, the statistical analysis of the data was performed using SPSS software (version 24.0) for Windows (IBM, Armonk, NY, USA). The samples were calculated on a heuristic basis. Averages ± SDs were used to present the data. In order to measure the variations in the binding ability of PCC with either oil, the Mann–Whitney U test was used in the tube test experiments. The alpha value was set at 0.05 for significance. The ‘pls’ model [11] was used to perform a partial least squares (PLS) analysis on the oil-binding capacity of chitosan. The DDA and degree of polymerization (DPw) were related to the oil-binding capacity using PLS. Initially, S1–S10 were used as the training set to create the PLS. Just two variables (DD and DPw) were needed to fit the training set using the kernel approach with the “plsr” function, and the data were scaled before analysis. Utilizing LOO to cross-validate the model, the RMSEP was 9.6% while using only one component and 9.1% when using two.

## 3. Results

### 3.1. Tube Test

**Figure 2:**
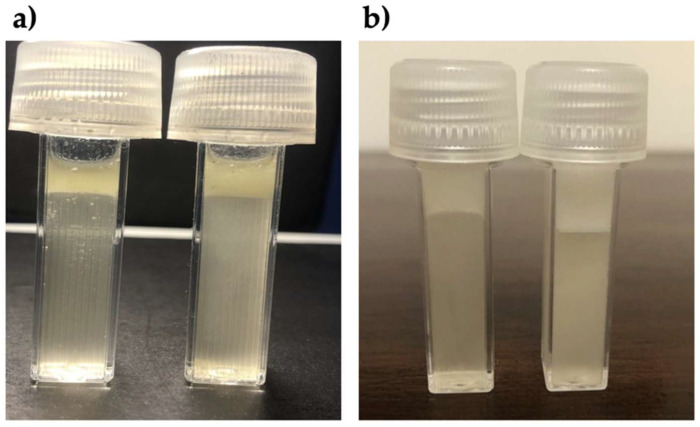
EVO and SO tube tests with or without PCC. (**a**) EVO Control tube on the left (5 cm emulsion) and PCC/EVO tube on the right (6.5 cm emulsion); (**b**) SO Control tube on the left (5 cm emulsion) and PCC/SO tube on then right (6.5 cm emulsion). The hydrogel values are shown in Table 1.

Figure 2 presents an example of the comparisons between oil Control tubes and oil tubes following PCC addition. According to the average of eight replicates (Table 1), the PCC/EVO binding was 1:11, whereas the PCC/SO bond was 1:15 (Mann–Whitney U test *p* < 0.05). Nevertheless, the difference between EVO and SO binding to PCC should be carefully addressed because of the very similar values.

This initial analysis, which involved the first measurement of suspension levels using an LCD, was conducted to readily assess the ability of chitosan to bind the oil. This method was not intended to replace more precise and well-established procedures, even though it can be helpful for a preliminary assessment. Actually, we used a more rigorous experimental approach in the final section of our study, using advanced analytical methods including a stereomicroscope and SEM to precisely and quantitatively evaluate the binding of fatty acids to chitosan. The LCD was simply utilized for a screening, and we ensured that the final outcome complied with accepted analytical guidelines for this type of research. We conducted the experiments properly in the lab, utilizing every necessary instrument and adhering to the suggested protocols in order to obtain accurate findings and ascertain the efficient formation of biofilms between PCC and the oils (EVO/SO).

### 3.2. Formation of PCC/Oil Aggregates in Laboratory Trials

**Figure 3:**
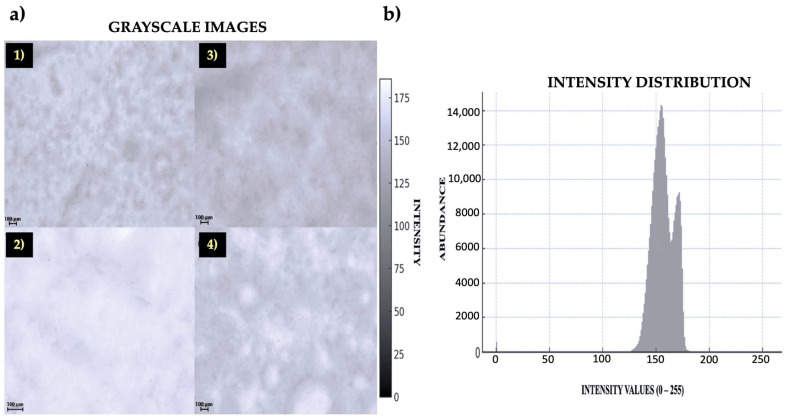
Stereomicroscope images of the hydrogels on glass microfiber filter (**a**) and contrast images to ascertain the intensity distribution of the aggregates generated (**b**). The hydrogel that developed between the PCC and EVO is depicted in (**a1**,**a2**), whereas the images of the hydrogel between the PCC and SO are shown in (**a3**,**a4**).

Stereomicroscope images were captured in order to further demonstrate the formation of emulsion/hydrogel (Figure 3, left panel). There were two phases (oil and hydrogel) with strong contrast, as shown by the peaks seen in the intensity distribution histogram (Figure 3, right panel).

### 3.3. Using PLS to Evaluate the Outcomes (Laboratory Trials)

**Figure 4:**
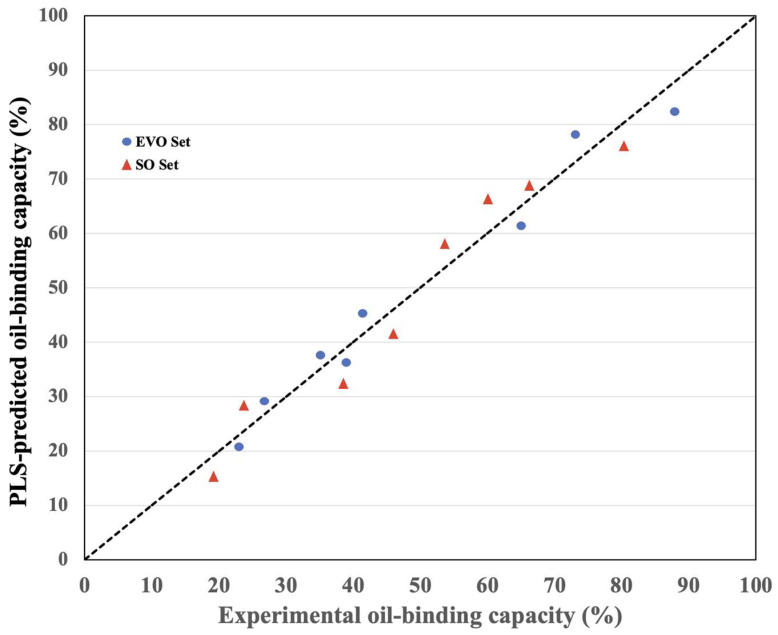
Predicting the oil-binding ability of chitosan using the partial least square (PLS) model has been externally validated. Experimental values = PLS − predicted values, as indicated by the dotted line.

The results from samples with low oil-binding capabilities were the sources of the significant prediction errors (Table 2). Figure 4 shows the relationship between the experimental and projected oil-binding capacities for the training and test sets using the PLS model. The internal validation result was consistent with the model’s prediction of the training set, as seen by the data points surrounding the line that reads experimental oil-binding capacity = anticipated oil-binding capacity.

A number of factors, both intrinsic to the chitosan–oil system and the experimental approach itself, can explain the variation in the SD values from the several samples displayed in Table 2. The degree of deacetylation, molecular weight, and particle size distribution of chitosan may vary from sample to sample. Two seemingly identical copies may behave differently because these variations directly impact the chitosan fibers’ or granules’ capacity to bind oil. Differences between SO and EVO include viscosity, the free fatty acid composition, and the presence of polyphenols in the case of EVO. The contact and anchoring on the chitosan surface are not completely repeatable in every replicate because of this difference in polarity and viscosity, which increases the standard deviation, particularly in EVO. Even when the coefficient of variation remains constant, the absolute SD tends to rise in tandem with the mean retention capacity value. Higher SDs are commonly seen with high means for the simple reason that there is more absolute volatility in µg or mg of oil. Improved control over the temperature, length, and speed of centrifugation and agitation may reduce this variability.

### 3.4. SEM Morphological Analysis (Laboratory Trials)

The SEM analysis enabled the morphology of hydrogel to be assessed. The scanning electron microscope had tungsten filament electron guns, which can operate between 0.5 and 30 kV and have a maximum resolution of 3.0 nm. Magnifications ranged from ×5 to ×50,000. It can operate run in low-vacuum modes with moist or non-conductive surfaces, and in high-vacuum modes for samples for the highest resolution. It has backscattered electron and secondary electron detectors (shaded, topographic, and composition). It features a five-axis asynchronous mechanical eucentric stage that can accommodates samples up to 20 cm in diameter, as well as an eucentric tilt and rotation. The SEM was fully computerized, operated on a computer, and automatically archived images in BMP, TIFF, or JPG formats.

**Figure 5:**
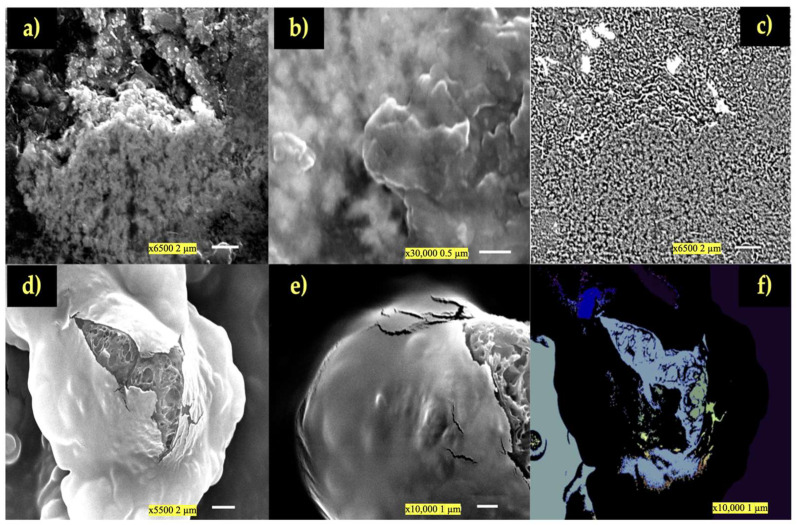
SEM images of the hydrogels formed between PCC and EVO (**a**–**c**) and between PCC and SO (**d**–**f**). Additionally, images with contrast enhancement are shown (**c**,**f**).

A well-formed hydrogel is indicated by the presence of a continuous, cross-linked structure in the SEM images. A homogeneous dispersion of oil droplets indicates that there is no coalescence, or fusion, of the drops. A smooth surface with fine porosity is another indication that PCC and the oil are interacting well. The SEM pictures of the hydrogels that developed between the PCC and oils are displayed in Figure 4, where it is observed a nice dispersion of the oil in the polymer matrix. The SEM images showed how PCC created a hydrogel network by revealing a spongy structure with pores left by water evaporation. The rate of drying has an impact on pore dispersion. Since drying was performed at ambient temperature, a slower rate will result in more uniform structure. If it is fast, more unequal pore formation will occur. SEM images may demonstrate smoother sections or aggregates and agglomerates with rough surfaces if the hydrogel had not been stable enough for the oil to separate before drying (Figure 5). The whitish patches in Figure 5b (binarized image) were likely oil droplets. There were not obvious clusters among the particles in Figure 5d, which appeared to be distributed quite equally. In the SEM images, the analysis found roughly 619 particles or structures (Figure 5d,e). The segmentation suggests the presence of discrete structures, which are typical of a mixture of particles and hydrogels and may be PCC-stabilized oil droplets or PCC particles. The SEM pictures acquired for this investigation clearly showed spherical oil droplets embedded in the PCC polymer matrix. A stable hydrogel with a homogeneous distribution and a somewhat uniform size (1–10 µm) was produced, as seen in Figure 5. If this hydrogel was unstable, we may observe phase separation or coalescence, which is the merging of larger droplets. The imprinting, or the presence of spheres or “circular imprints”, can be observed in Figure 5a,b. This suggests that the oil was well emulsified prior to drying. The particle size distribution is depicted by the histograms in Figure 5, where the majority of the particles fall within the submicron range. The average values of droplet size, as determined by area and particle diameter, are shown in Figure 5. The droplets’ average area was around 588.3 square pixels (Figure 5c). The typical size of the oil droplets embedded in the chitosan and/or in pores of the matrix is shown in this image. If these structures are round and well-spaced, as they were in this study, it is because the oil droplets were well distributed before drying. With an average intensity of 152.7 (on a scale of 0–255) and a standard deviation of 69.7, the SEM image analysis revealed a good contrast between the backdrop and the current structures (Figure 5c). The particles in the SEM image had an average diameter of 0.18 µm, a median diameter of 0.09 µm, and a standard deviation of 0.69 µm, according to the granulometric analysis of the particles (Figure 5f). These findings imply the existence of PCC–oil microparticles, whose size varied according to the method of preparation (i.e., level of emulsification or homogenization).

The data unmistakably display a narrow, centered distribution with a low standard deviation, suggesting that the hydrogel was stable and constant in both scenarios and that the droplets or pores were fairly uniform in size. However, there would have been a range of small and large pores or oil droplets of different sizes if the KDE curve had been broad or had several peaks.

### 3.5. In Vitro Fat-Binding Capacity (Laboratory Trials)

In general, the fat-binding capacity is the quantity of oil that a polymer sample (i.e., PCC) can hold on to per unit mass of a dry sample. The unit of measurement is g oil/g sample [5]. The pK_a_ of PCC is 6.3. Nearly all of the amines in PCC are charged (NH_3_^+^) at lower pH values, which enhances its hydrophilicity, dispersion in solution, and capacity to form weak contacts (both hydrophobic and electrostatic) with oil droplets. While SO acidity levels vary from 0.9 to 1.1 mg of KOH/g of oil, EVO acidity values range from 0.8 to 2.0 mg of KOH/g of oil. This contact mechanism between PCC and fat molecules can be explained by the fact that PCC is a weak cationic polyelectrolyte that can hydrate and disperse in acidic aqueous solutions. PCC molecules exhibit a high charge under these conditions due to the protonation of the amino groups in the polymer chains. Micelles and fat emulsions are generated when negatively charged molecules and particles, such as cholesterol, fatty acids, bile acids, and triglyceride droplets, are attracted to the ionized polycationic form of PCC. The deprotonation of amino groups in neutral or alkaline aqueous conditions (pH > 6.5) causes polycationic PCC to lose its charges when it enters the duodenum, which results in the formation of micelles and fat emulsions encircled by chitosan [5].

**Figure 6:**
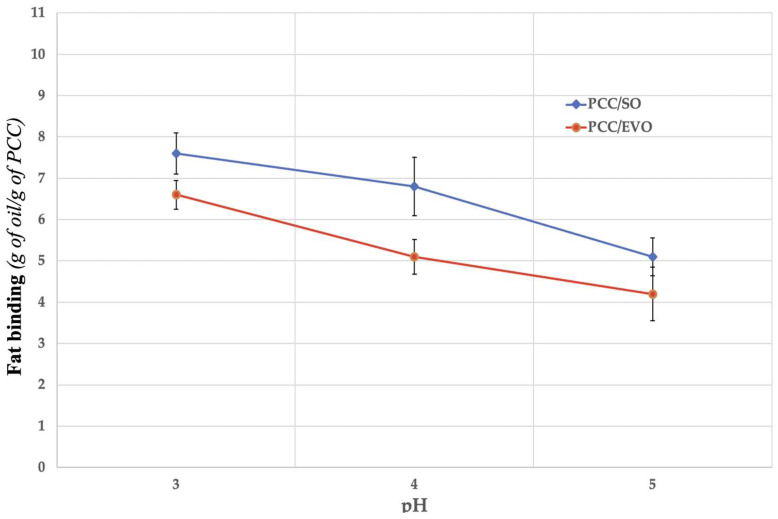
Fat-binding capacity of PCC for EVO/SO at different pH values.

Figure 6 exhibits how the fat-binding capability of the EVO and SO PCC samples varies with pH. Samples with pH values of 4 and 5 contained NaHCO_3_ in order to replicate the rise in pH in the duodenal tract that occurs when partially digested food moves from the stomach into the small intestine.

Similar acidity values have been reported for these fatty materials in the literature [5,12]. PCC interacts with and traps lipids because it is soluble in acidic media (stomach pH) and semi-soluble and/or insoluble in basic or slightly acidic media (duodenal pH). Figure 6 indicates that the fat-binding values were comparatively high in both PCC/SO and PCC/EVO scenarios at pH 3, at 7.6 and 6.6, respectively. For PCC with good lipid affinity, these values fell within the usual range. The fat-binding properties of PCC/SO and PCC/EVO differed significantly when the pH rose to 4. The fat-binding value of PCC/SO was still high, having decreased by just about 11%. The PCC/EVO fat-binding score, on the other hand, decreased by almost 23%, suggesting a moderate capacity for fat binding. PCC/SO lost an additional 22% at duodenal pH (pH 5), resulting in a rather low fat-binding value of 5.3. Nevertheless, a fairly low value (4.2) was obtained in the PCC/EVO scenario, when a further 18% reduction in fat binding took place. Both oils demonstrated their maximum capacity to bind fat at a pH of 3. As would be expected given the loss of PCC protonation, the fat-binding efficacy gradually decreased as the pH rose. All samples were able to trap fat molecules (Table S1).

## 4. Discussion

Despite previous studies [2,3,13], the in vitro binding of chitosan with lipids has limitations because, unlike our work, not all biological factors can be taken into consideration. Since the mixture of chitosan and fat is “frozen” in a sort of gel after the bulk reaches the duodenum and ileum due to the pH rise and the limited number of new bindings, the stomach has a major impact on chitosan activity in terms of lipid binding [13,14]. Under light of this, we sought to ascertain what would happen with chitosan’s lipid-binding activity under situations that mimic stomach digestion. Reducing body weight requires consuming less calories from intermediate and long-chain FAs [10]. This investigation only looked at edible oils in the C12 (lauric acid) and C20 (arachidonic) range, since the percentages of SAFAs, MUFAs, and PUFAs were 14.7/73/7.5 in EVO and 11/34/50 in SO, respectively (https://www.ncbi.nlm.nih.gov/books/NBK570127/ (accessed on 10 January 2025)). The finding that the binding with PCC appears objectively more pronounced for SO (Table 1) is consistent with a higher concentration of polar lipids in this oil, among which PUFAs are more significantly prevalent than in EVO. Even while the action of enzymes (especially lipases) that totally hydrolyze trioleins could not be taken into account, the few enzymes of this class present in the oil itself [15,16], in conjunction with mechanical coarctation (agitation), might release FAs from their bond with glycerol. Finally, the interplay of the gastric components in creating networks connecting chitosan, lipids, and starch gives support to our working hypothesis [17]. Together with the presence of acidic dietary ingredients (ascorbic and tartaric acids), starch binds chitosan to create a variety of small networks in the stomach [18] that aid in the excretion of dietary fat [19]. All the evidence suggests that chitosan can form lipid linkages through gastric digestion and not only in the artificially induced environments used in chemical experiments.

Among the chemical–physical phenomena that can happen when PCC is combined with EVO or SO at pH 3 are (1) the solubility of chitosan in an acidic aqueous medium, (2) the interactions with oil (PCC forms a colloidal dispersion or nanoemulsion with oil, especially if stirring is used), and (3) the formation of a hydrogel in which PCC assumes a positive charge because of the amino groups (-NH_2_ → -NH_3_^+^) [20,21]. This characteristic may promote electrostatic contact with certain polar oil constituents, such polyphenols or phospholipids. A stable hydrogel emulsion, also known as the “milky hydrogel”, is created when the mixture is vigorously agitated. After a few hours, if there is no oil separation and the hydrogel is stable [20,22], oil viscosity and the triglyceride content have favorable impacts on the stability of the system [23,24]. Actually, SO increases the stability of the hydrogel (see Table 1) by having a higher concentration of PUFAs, such as linoleic acid, and a lesser concentration of polar molecules. EVO has more polyphenols and bioactive substances, as well as MUFAs, such as oleic acid. EVO hydrogel cross-linking can be improved by the interactions of polyphenols with PCC via electrostatic and hydrogen bonding forces [24,25].

**Figure 7:**
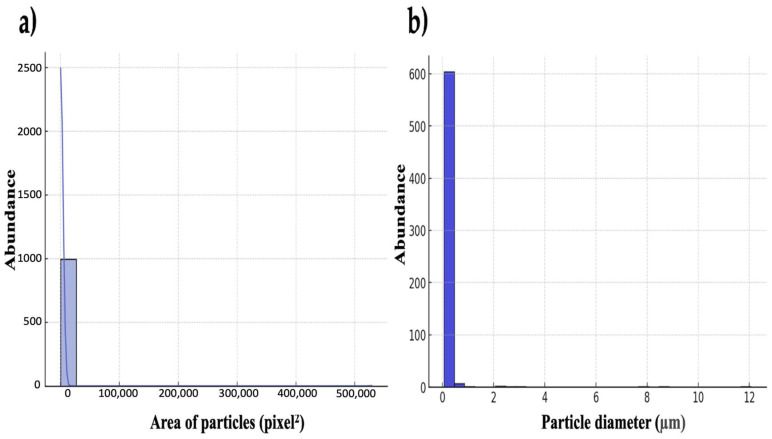
Histogram of the droplet size distribution (area and diameter). The area of the droplets or pores (in pixels^2^) is shown on the X-axis (**a**). The frequency with which objects of a given size appear is displayed on the Y-axis. The Kernel Density (KDE) curve is the smooth line that helps visualize the data distribution (**b**).

A number of chemical forces and molecular interactions affect the development of the PCC hydrogel with oil in an acidified aqueous environment, including hydrogen bonds, electrostatic interactions, van der Waals forces, and hydrophobic forces. Hydrogen bonds affect how the network interacts with water—for example, between the hydroxyl and amino groups of PCC, between PCC and water molecules when the network is hydrated, and between PCC and polyphenols in the case of EVO [24,25]. Electrostatic interactions are necessary for PCC to gel in an acidic environment. PCC is a cationic polymer, which means that it has a positively charge at pH 3 because of the protonation of its amino groups, thus interacting with existing anions (i.e., free FAs, charged phospholipids as in the case of EVO, and surfactants). Between the PCC polymer chains are van der Waals forces, which aid in the self-assembly of the hydrogel and contribute to the cohesiveness of the structure, as seen in Figure 5 [23,26,27]. van der Waals forces are also present between the polymer matrix and scattered oils with longer, less polar lipid chains, such as EVO. As to hydrophobic interactions, the stability of the hydrogel may be impacted by weaker interactions that arise from an oil’s higher unsaturated fatty acid (i.e., like SO) content. Lastly, covalent interactions that serve as cross-linking agents and reinforce the hydrogel network must be taken into account if they are present. Aldehydes or polyphenols may react (i.e., covalently cross-linked with glutaraldehyde or tannins), and PCC may undergo chemical changes (i.e., oxidation or cross-linking with other polymers) [23,26,27]. The potential interactions between PCC and oil triglycerides in the stomach environment are schematized in Figure 7. The viscosity, mechanical resistance, and structure of the hydrogel would all be significantly altered as a result of lipid oxidation. Aldehydes and ketones, high peroxide numbers, and free radicals could react with PCC and change the cross-linking, reducing or changing its molecular structure, cohesiveness, and hydrogel stability [28,29].

The SEM morphological analysis showed an even distribution of the hydrogel particles (Figure 4), suggesting that the phases may be gradually transitioning or well mixed [30,31]. If the hydrogel had not been homogenized, as indicated by deformed or fused drops, or if there was an unstable hydrogel with isolated drops without a polymeric network, the SEM analysis could have revealed a demulsion or increased instability of the hydrogel [32,33,34]. The hydrogel created by the union of PCC and EVO performed better than the polymer matrix that came from PCC and SO [35,36]. The enhanced SEM images and contrast analysis (Figure 5) showed that the surface appeared more porous in the EVO example, where the PCC produced a better three-dimensional network that was helpful for encapsulating the oil [35,36,37].

Another feature taken from SEM images is the size of the oil droplets (Figure 5b). If they are excessively large, they indicate a potential phase separation and reduced stability. Although integration is improved when the droplets are too small, the viscosity of the hydrogel may decrease [23,37]. Figure 5 shows that the distribution in this instance was narrow and concentrated around a specific value, indicating that the size of the droplets or holes was reasonably uniform [35,36,37].

**Figure 8:**
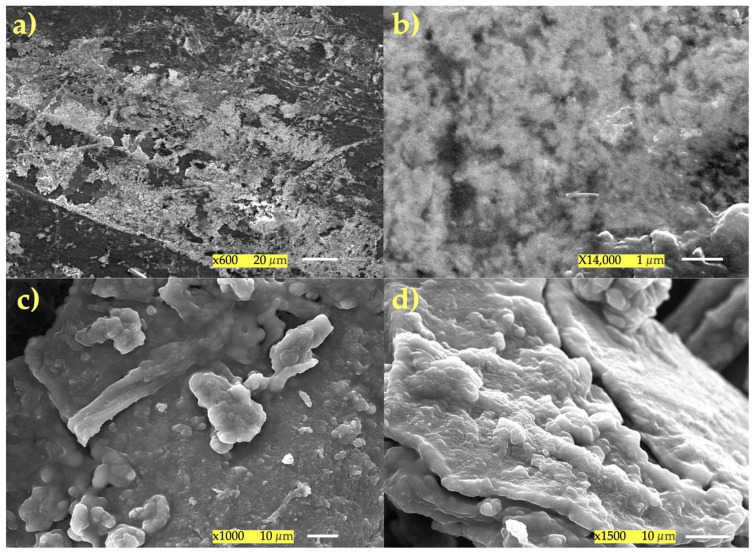
SEM images of the polymer–lipid framework between PCC and EVO (**a**,**b**) and between PCC and SO (**c**,**d**).

These SEM images in Figure 8 clearly demonstrate that without the presence of enzymes, a polymer–lipid framework between PCC and oils (EVO—Figure 8a,b and SO—Figure 8c,d) forms at pH 3, or under conditions that are quite similar to the gastrointestinal environment. Figure 8a,b depict PCC with EVO, which appeared to be a very heterogeneous matrix continuum with dense (“massive”) PCC zones that were cross-linked by surface pores and rougher regions (Figure 8a). The oil droplets stabilized by hydrogen bonding and electrostatic interactions with the PCC network are reflected by these properties, which are surfactant deposits. Figure 8b displays tiny spherical or hemispherical aggregates that are securely attached to the polymer on the surface of the network. The polymer encircled and stabilized the oil, creating a real interfacial network. These structures are equivalent to microdroplets of olive oil coated or “encapsulated” by thin sheets of PCC. The consistency of the polymeric matrix and the existence of these embedded microdroplets demonstrate the formation of a solid hydrogel: the oil was trapped in a colloidal network of PCC, which, at an acidic pH, gains a positive charge and attaches to the lipid chains via weak bonds and hydrophobic attraction.

The interaction between SO and PCC is illustrated in Figure 8c,d. Figure 8c shows PCC forming irregular plates (or “sheets”) that are tens of microns thick, with granular clusters that are 1–2 µm in diameter, and microfibrils defining pores and corridors. These clusters are SO aggregates connected by tiny PCC bridges. In Figure 8d, lipid spheres and thin flakes are positioned between stacked layers of PCC that resemble “onion slices”. The surface still has a rough texture due to the physicochemical cross-linking between the PCC chains and the sunflower triglycerides. The oil droplets are immersed within a three-dimensional PCC framework, as evidenced by the lamellar shape and the even distribution of lipid spheres. The formation of polymer–lipid bridges visible on the rough, porous surface indicates that the oil is naturally encapsulated and stable at pH 3 without the use of enzymes. All of the oil droplets are immersed in the PCC matrix, forming a stable and continuous network. None of the SEM images display free oil droplets or macroscopic aggregates. A physicochemical (hydrophobic and electrostatic) polymer–oil interaction is evident from the lipid aggregates rough texture, spherical form, and strong adherence to the PCC. Chitosan is given a positive charge by the protonation of it at pH 3 (NH_3_^+^), which promotes the spontaneous formation of this emulsified network. This implies that the gastrointestinal environment may be replicated by pH adjustment alone, without the need for enzymes. These findings suggest that a PCC–oil emulsified network will probably emerge, which may be helpful for gastric release systems.

According to in vitro test of PCC fat binding, it can be stated that a PCC layer surrounding the fat emulsion droplet can restrict the amount of fat absorbed by reducing lipid absorption in the duodenum and blocking lipases from accessing the PCC-surrounded lipid substrate. The PCC–lipid complex is precipitated when the polycationic PCC loses its positive charge as a result of the deprotonation of amino groups in neutral and alkaline solutions [5]. The results support the notion that a high-DDA PCC is better at capturing fat droplets because the polymer chain has more free amino groups and positive charges, which strengthen the electrostatic interactions between PCC and negatively charged lipid molecules. Under acidic conditions, PCC dissolves and produces a gelatinous structure that can hold on to fat droplets. The ability of irradiated and sonicated chitosan samples with a molecular weight ranging from 25 to 408 kDa and a DDA of roughly 88% to bind fat was evaluated by Czechowska-Biskup et al. [38]. Through hydrophobic interactions, PCC can fill the void created by the volume of unsaturated fatty acid molecules. However, because they are compressed, saturated fatty acids might not interact as much with PCC. Compared to a less acidic environment (pH = 5), these characteristics helped PCC retain more SO and EVO in a more acidic environment (pH = 3). PCC chemistry states that oil binding works better at pH 3 than pH 5. As seen in the SEM pictures in Figure 5 and Figure 8, the polymer expands at pH 3, when PCC is nearly fully charged, forming a highly porous network that effectively captures oil micro-droplets. PCC is less soluble at the duodenal pH (pH 5), part of the network breaks down, and the stability of the solid hydrogel and contact surface are reduced. This structure implies that PCC’s ability to bind fat at higher pH values is decreased because the amino groups have a weaker positive charge. The protonation of amino groups in PCC is mainly the primary cause of the decline in fat binding with rising pH. The highly protonated amino groups (-NH_2_) of PCC at pH 3 have a larger positive charge and are more likely to interact electrostatically with the polar groups in EVO/SO. The partial deprotonation of these groups at less acidic pH (i.e., 4–5) lowers the positive charge and weakens the interactions with the oil. PCC hydrogel synthesis (cross-linking) with EVO/SO works better at acidic pH levels. A higher pH makes the hydrogel’s structure less stable or flexible, which reduces its ability to bind oil. The final consideration is PCC’s solubility. PCC dissolves in acidic media. The loss of solubility of PCC and the capacity to create stable hydrogels at pH values ≥ 5.0 adversely impacts fat retention (Figure 6). Due to the lower degree of unsaturation of the EVO—SO includes more polyunsaturated linoleic acid, while EVO is predominantly composed of monounsaturated oleic acid—PCC/EVO fat-binding values are lower than those of PCC/SO. Polyunsaturated oils have more functional groups that can interact with PCC, especially at higher pH values when electrostatic interactions diminish. Furthermore, even at higher pH values, SO and PCC combine to generate more stable hydrogels and emulsions, which enhance oil retention. Even if SO is partially deprotonated, it has a better affinity for the PCC gel because it contains a higher percentage of triglycerides with more polar chains.

**Figure 9:**
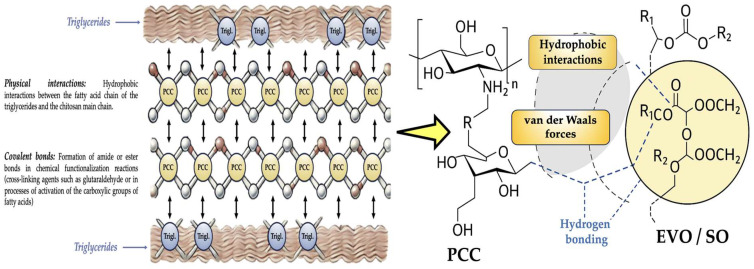
Chemical network between chitosan and triglycerides (EVO/SO) in the stomach.

Our study is the first investigation into how the hydrogel between chitosan and the two types of oils is generated at an acidic pH (which mimics the gastric environment), as well as how the morphological structure of the hydrogel can be used to determine how the oils and chitosan were distributed and how the reticulum was formed (Figure 9).

## 5. Putative Limitations of This Study

The food type, postprandial time, and hydrochloric acid secretion are some of the factors that affect gastric pH, which varies during digestion between 1.5 and 3.5. Throughout the current study’s trials, a pH of 3.0 was maintained as a typical experimental condition, which gives an average value in the physiological pH range of the stomach during the early postprandial period. According to previous studies, the pH of the stomach tends to settle at roughly 3.0 during the digestion phase when partially buffered foods are present, particularly in mixed meals [39,40]. As a result, using a pH of 3.0 as the experimental value reduced variability, facilitated comparability with prior research, and offered a physiologically realistic intermediate condition. Furthermore, rather than simulating the whole digestion or pH progression along the gastrointestinal tract, the purpose of this study was to evaluate the durability and behavior of hydrogel/biofilm formation under settings of average gastric acidity. This study was not intended to account for other stages of digestion, such as the transition to the duodenum, where pH rises to values close to 5–6. The use of a pH that is neutral or slightly acidic (such as the pH of the duodenum of 5) was ignored due to these circumstances. The degree of protonation of the amino groups of chitosan drastically decreases, which lowers the solubility of the substance in the medium, as well as its capacity to form complexes or networks with lipophilic substances. The efficient development of aggregates, gels, or networks between chitosan and lipids occurs primarily under acidic conditions (pH < 4.5), which is congruent with the physiology of the stomach rather than the small intestine or duodenum.

The use of gastric enzymes, such as lipases, was purposefully avoided, in this investigation, since the primary objective of this study was to evaluate biofilm formation resulting from the interaction between chitosan and vegetable oils (EVO and SO) under conditions that mimicked the acidity of the gastric environment. The physicochemical principle states that the generation of biofilms in our system is determined by the electrostatic and hydrophobic interactions between PCC and the triglycerides in the oils. Since the oil has not yet undergone significant hydrolysis, these interactions took place during the early phases of contact. Similar prior research indicates that the action of lipases, which catalyze the hydrolysis of triglycerides into free fatty acids and monoglycerides, does not substantially alter the early stages of biofilm aggregation and development [41]. Therefore, we feel that their absence enhanced the isolation and investigation of the phenomenon under study rather than undermining the veracity of the conclusions gained about the mechanisms of biofilm growth.

The form and distribution of particles in chitosan biofilms have been evaluated in previous studies [42,43], which often utilize ImageJ to measure aggregation. Comprehensive details on the number of fields examined in the SEM analysis, as well as statistical measures of variability were provided in Supplemental Material (Figure S2). Additional controls, such as oil samples devoid of chitosan and chitosan samples, were added to our inquiry in order to distinguish between specific interactions and generic aggregation processes in acidic environments. All these experiments were carried out under the same experimental conditions. The SEM images of the control samples differed significantly from those ones containing both oil and chitosan, suggesting that specific interactions between these two chemicals were responsible for the observed aggregation (Figure S2).

Advanced characterization methods like Fourier transform infrared spectroscopy (FTIR) were judged unrelated to our aims. The aim of the present study was functionality. Although the FTIR technique is useful for identifying functional groups and characterizing polymeric materials, it was not necessarily required in this case. This is because the general chemical structure of chitosan is widely known, and the biopolymer did not undergo additional chemical modifications that would necessitate a spectroscopic recheck. Finally, the selection of simpler and more straightforward approaches also addressed concerns regarding analytical efficiency and resource availability by choosing instruments that produced unambiguous, repeatable results directly related to our hypothesis without adding needless analytical complexity.

## 6. Conclusions

Our investigation is the first study where the oil-binding capacity in an acidic aqueous environment (pH = 3) was analyzed as a measure of the strength of the retention of EVO and SO by chitosan (DDA > 90%) in the stomach media. The SEM morphological analysis demonstrated that their combination produced homogeneous hydrogels as evidence. Based on the observed binding efficiency, chitosan’s high degree of deacetylation strengthens its capacity to interact hydrophobically and electrostatically with lipids, supporting a possible role in regulating fat absorption and digestion. Our findings offer fresh perspectives on the behavior of chitosan in the gastrointestinal tract and underscore its uses in food formulations, dietary fat control, and pharmacology pertaining to lipid metabolism. Nevertheless, the mechanisms behind this interaction and its consequences in vivo require more investigation.

## Figures and Tables

**Table 1:** EVO and SO (0.3 g/mL) hydrogel levels in the tube test with or without PCC (1.6 mg/mL) at pH 3.

Replicate #	Oil			
	EVO		SO	
	Weight (g)	Height (cm)	Weight (g)	Height (cm)
1	12.5	6.0	18.8	6.5
2	12.5	5.0	12.5	6.0
3	6.2	5.5	12.5	6.0
4	12.5	6.0	12.5	6.0
5	12.5	6.0	12.5	6.0
6	6.2	5.5	12.5	6.0
7	12.5	6.0	18.8	6.5
8	12.5	6.0	18.8	6.5
Mean	11.0	6.0	15 *	6.0
SD	2.9	0.2	3.3	0.3

Mann–Whitney U-test: SO vs. EVO (* *p* < 0.05).

**Table 2:** Oil-binding capacity (%) of the PCC samples (laboratory trials).

Replicate #	EVO		SO	
	Value	SD	Value	SD
1	21.7	2	19.4	3
2	27.7	4	24.2	5
3	36.5	5	34.6	5
4	39.3	4	44.2	4
5	41.1	5	54.3	5
6	62.8	4	60.1	4
7	74.8	6	69.7	6
8	86.6	6	78.9	5

## Data Availability

The original contributions presented in this study are included in the article/Supplementary Material. Further inquiries can be directed to the corresponding author.

## Supplementary Materials

The following supporting information can be downloaded at https://www.mdpi.com/article/10.3390/polym17111445/s1: Figure S1. Chemical characteristics of chitosan and certificate; Table S1. Fat-binding capacity of PCC with EVO/SO at different pH values; Figure S2. SEM images of PCC without oils (a,b), PCC with EVO (c,d), and PCC with SO (e,f).

## Abbreviations

The following abbreviations are used in this manuscript:

DDA	Degree of deacetylation
DPw	Degree of polymerization
EVO	Extra virgin oil
FA	Fatty acids
FBC	Fat-binding capacity of chitosan
FIA	Food intake assessment
FTIR	Fourier transform infrared spectroscopy
LCD	Precision digital caliper
MMW	Medium molecular weight
MUFAs	Monounsaturated fatty acids
MW	Molecular weight
PCC	*Procambarus clarkii* chitosan
PLS	Partial least squares
PUFAs	Polyunsaturated fatty acids
SAFAs	Saturated fatty acids
SEM	Scanning electron microscope
SO	Sunflower oil
