# Supplementary material for: Enhanced Oil Binding Potential of Procambarus clarkii Chitosan (PCC): A Study with Extra Virgin Olive Oil and Sunflower Oil Under Simulated Gastric Conditions
Source: Polymers (Basel). 2025 May 23;17(11):1445. doi: 10.3390/polym17111445 (PMC12157860; doi:10.3390/polym17111445)

Supporting Materials

Revolutionizing Enhanced Oil Binding Potential of *Procambarus clarkii* Chitosan (PCC): A Comparative Study with Extra Virgin Olive Oil and Sunflower Oil under Simulated Gastric Conditions

Claudio Casella[1], Umberto Cornelli[2], Santiago Ballaz[3], Giuseppe Zanoni[1] and Luis Ramos-Guerrero[4*]

[1*] Department of Chemistry, University of Pavia, Pavia, (Italy)

[2] School of Medicine, Loyola University, Chicago (USA)

[3] Faculty of Heath Sciences, Universidad del Espíritu Santo, Samborondón, (Ecuador)

[4] Grupo de Investigación en Bio-Quimioinformática, Carrera de Ingeniería Agroindustrial, Facultad de Ingeniería y Ciencias Aplicadas, Universidad de Las Américas (UDLA), Quito, (Ecuador)

Figure S1. Chemical characteristics of chitosan and certificate

Nutratrade
YOUR TRUST IS OUR SUCCESS®

**NUTRATRADE S.r.l.**			**TECHNICAL DATA SHEET**		
Name **Chitosan**			SOURCE: **PROCAMBARUS CLARKII**		
Issued by	*QA*	Approved by	*Riccardo Cultraro*	Date **15/09/2023**	Rev. **5**

**Product Name**	Chitosan	**Solvent Used**	NA
**Country Origin**	China	**Grade**	Food ☒ Feed ☐ Pharma ☐
**CAS No.**	9012-76-4	**Molecular Formula**	$C_{56}H_{103}N_9O_{39}$
**Part No.**	4.010-126.0	**Shelf life**	24 months when properly stored
**Packing**	Paper-drums and two plastic-bags inside, food contact material compliant with EC Reg. 1935/2004 and EU Reg. 10/2011	**Storage**	Store in cool & dry place. Do not freeze. Keep away from strong light and heat.

**Analysis Item**	**Specification**	**Method**	**Provided on delivery**
Deacetylated Degree	>90%	NA	CoA
DER	NA	NA	NA

**Chemical and Physical Characteristics**			
**Analysis Item**	**Standard value**	**Method**	**Provided on delivery**
Appearance	Powder	Visual	DoC
Color	White to light yellow	Visual	DoC
Odor& Taste	Characteristic	Organoleptic	DoC
Identification	Positive	NA	DoC
Excipient	NA	NA	NA
Loss on drying	≤10.0%	Ph.Eur.9.0<2.2.32>	CoA
Total ash	< 1%	Ph.Eur.9.0<2.4.16>	CoA
Solubility in Water	NA	Organoleptic	DoC
Solubility in oil	NA	NA	DoC
Apparent density	NA	Eur.Ph.<2.9.34>	CoA
Tapped density	NA	NA	CoA
Solubility((in 1% Acetic Acid))	> 99.0%	NA	CoA
Viscosity	90-130 mPa·s(cP)	NA	CoA
Sieve analysis	95% through 100 mesh	USP39<786>	DoC
Arsenic (As)	NMT 1 ppm- Reg.EU 2023/915	Ph.Eur.9.0<2.2.58>ICP-MS	DoC
Cadmium (Cd)	NMT 1 ppm- Reg.EU 2023/915	Ph.Eur.9.0<2.2.58>ICP-MS	DoC
Lead (Pb)	NMT 3 ppm- Reg.EU 2023/915	Ph.Eur.9.0<2.2.58>ICP-MS	DoC
Mercury (Hg)	NMT 0.1 ppm - Reg.EU 2023/915	Ph.Eur.9.0<2.2.58>ICP-MS	DoC
Heavy Metals	NMT 10 ppm- Reg.EU 2023/915	Ph.Eur.9.0 <2.4.8>	DoC
Pesticides Residues	Conform Reg.(EC) .396/2005 and amendments.	Gas Chromatography	DoC
Solvent Residues	Conform Eur.Ph. 9.0 <5,4 > and EC Dir. 2009/32	Ph.Eur.9.0<2.4.24>	DoC
Hydrocarbons PAHs	≤ 50 ppb -Reg.EU 2023/915	GC-MS	DoC
Benzo(a)pyrene	≤ 10 ppb -Reg.EU 2023/915	GC-MS	DoC
Radioactivity	≤ 600 Bq/Kg -Reg. EC 1048/2009	NA	DoC
Aflatoxin B1	≤ 5 ppb - Reg.EU 2023/915	Ph.Eur.9.0<2.8.18>	DoC
Aflatoxins Σ B1, B2, G1, G2	≤ 10 ppb - Reg.EU 2023/915	Ph.Eur.9.0<2.8.18>	DoC

Nutratrade
YOUR TRUST IS OUR SUCCESS®

**Microbiological Characteristics**			
Total aerobic Count	≤1000 cfu/g	Ph.Eur.9.0<2.6.12>	DoC
Yeast/Moulds	≤100 cfu/g	Ph.Eur.9.0<2.6.12>	DoC
Enterobacteriaceae	≤100 cfu/g	Ph.Eur.9.0<2.6.31>	DoC
Escherichia coli	Absent in 1 g	Ph.Eur.9.0<2.6.31>	DoC
Salmonella spp.	Absent in 25 g	Ph.Eur.9.0<2.6.31>	DoC
Listeria monocytogenes	Absent in 25 g	Ph.Eur.9.0<2.6.31>	DoC
Staphylococcus aureus	Absent in 1 g	Ph.Eur.9.0<2.6.31>	DoC

**Other product characteristics**		
Irradiation	No Irradiation	DoC
GMO	Product No-GMO (Reg.1829/2003-1830/2003 EC)	DoC
Allergens	Crustaceans products thereof: Chitosan	DoC
Food Additives	Free (Reg. EU 1333/2008 and amendments)	DoC
BSE/TSE	Free	DoC
Melamine	Free, No melamine (Reg.EU 2023/915)	DoC
Pyrrolizidine alkaloids	In compliance with Reg.EU 2023/915	DoC
Nanomaterials	Absent in compliance with Reg CE 1169/2011	DoC
Ethylene oxide	No Ethylene oxide	DoC
Suitable for Vegan	No	DoC

*DoC-Declaration of Conformity
*CoA-Result on CoA

Table S1. Fat-binding capacity of PCC with EVO/SO under different pH values

		**PCC/SO**	
**N. Sample**	**Fat-binding value (pH=3)**	**Fat-binding value (pH=4)**	**Fat-binding value (pH=5)**
1	8.1	6.7	5.8
2	7.5	7.6	4.9
3	7.1	6.2	5.2
**Mean**	**7.6**	**6.8**	**5.3**
**SD**	**0.5**	**0.7**	**0.5**
		**PCC/EVO**	
**N. Sample**	**Fat-binding value (pH=3)**	**Fat-binding value (pH=4)**	**Fat-binding value (pH=5)**
1	6.9	5.4	4.9
2	6.6	5.2	3.7
3	6.2	4.6	3.9
**Mean**	**6.6**	**5.1**	**4,2**
**SD**	**0.4**	**0.4**	**0.6**

Figure S2. SEM images of PCC without oils (a, b), PCC with EVO (c, d) and PCC with SO (e, f)

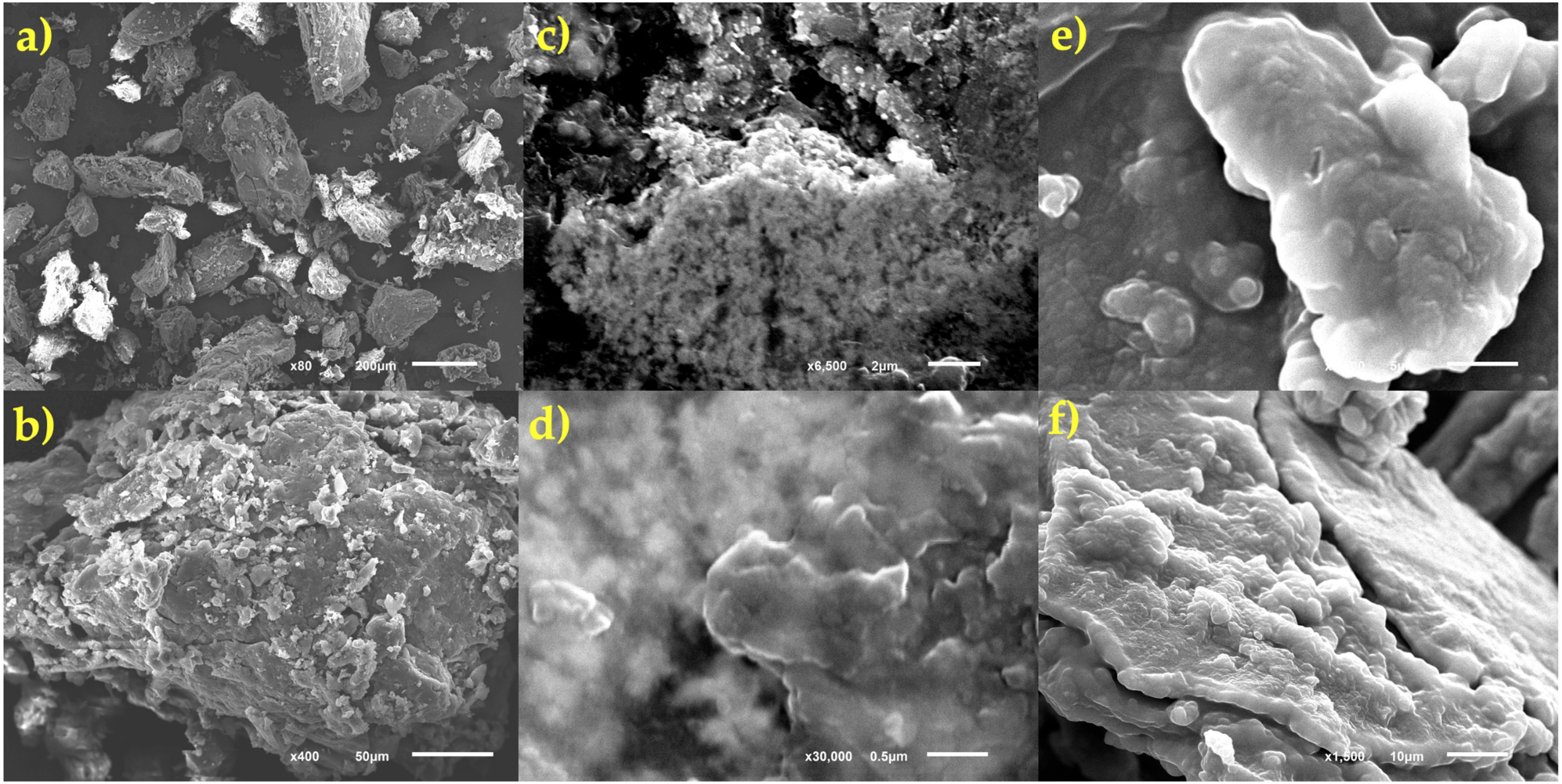

Supplement: Supplementary file 1 [file polymers-17-01445-s001.zip › polymers-3663314-supplementary.pdf]
